# ASO Author Reflections: Routine Omentectomy During Cytoreductive Surgery and HIPEC

**DOI:** 10.1245/s10434-022-12775-8

**Published:** 2022-11-11

**Authors:** Sohini Khan, Joel M. Baumgartner

**Affiliations:** Division of Surgical Oncology, Department of Surgery, University of California San Diego, 3855 Health Sciences Dr. #0987, La Jolla, CA 92093-0987 USA

## Past

The omentum is a common site of metastasis encountered in patients with peritoneal metastases.^1^ Although the goal of cytoreductive surgery (CRS) followed by hyperthermic intraperitoneal chemotherapy (HIPEC) is to remove all gross peritoneal disease, there is no standardized protocol.^2^ Hence, although omentectomy is commonly performed,^3^ variability exists in whether greater omentectomy is performed even in the absence of visible omental metastases. We wanted to determine the incidence of occult histologic omental metastasis (OHOM), rate of omental recurrence in those who did not undergo omentectomy, and morbidity associated with undergoing omentectomy in this study.

## Present

Greater omentectomy was performed in the vast majority—84.9%—of the 683 CRS-HIPEC procedures evaluated at our institution. Most resected omentums (77.9%) had gross intraoperative evidence of omental metastases. However, a relatively high proportion of resected omentums (31.9%) that appeared grossly normal had evidence of OHOM, although the majority of these cases included acellular mucin within the omentum. Of patients who did not undergo omentectomy and had confirmed documentation of a residual omentum, 55.8% developed recurrent disease after a median follow-up of 25.9 months with over one-third of recurrences involving the omentum. Omentectomy was not associated with an increased rate of overall morbidity measured by the 60-day Comprehensive Complication Index or delayed gastric emptying.

## Future

Our study highlights the rate of occult metastases within the omentum in patients undergoing cytoreduction for peritoneal metastases, the lack of demonstrable increased morbidity with performing omentectomy, and the relatively high rate of occurrence within the omentum when it is left in place. These findings argue in favor of performing routine omentectomy during CRS-HIPEC procedures. However, the biologic propensity for omental metastases, implications of acellular mucin within the omentum, and the influence of omentectomy on overall survival remain areas of uncertainty and warrant further investigation.

## References

[1] Gerber SA, Rybalko VY, Bigelow CE (2006). Preferential attachment of peritoneal tumor metastases to omental immune aggregates and possible role of a unique vascular microenvironment in metastatic survival and growth. Am J Pathol.

[2] Morano WF, Khalili M, Chi DS, Bowne WB, Esquivel J (2018). Clinical studies in CRS and HIPEC: trials, tribulations, and future directions–a systematic review. J Surg Oncol.

[3] Koppe MJ, Nagtegaal ID, de Wilt JH, Ceelen WP (2014). Recent insights into the pathophysiology of omental metastases. J Surg Oncol.

[4] Khan S, Nguyen-Huong D, Mojgan H, Kelly KJ, Veerapong J, Lowy AM, Baumgartner JM (2022). Is routine omentectomy a necessary component of cytoreductive surgery and HIPEC?. Ann Surg Oncol.

